# Cyclooxygenase-2 Inhibition Reduces Autophagy of Macrophages Enhancing Extraintestinal Pathogenic *Escherichia coli* Infection

**DOI:** 10.3389/fmicb.2020.00708

**Published:** 2020-04-17

**Authors:** Haiyan Ren, Xuhua Chen, Fengwei Jiang, Ganwu Li

**Affiliations:** ^1^Department of Veterinary Preventive Medicine, College of Veterinary Medicine, Nanjing Agricultural University, Nanjing, China; ^2^State Key Laboratory of Veterinary Biotechnology, Harbin Veterinary Research Institute, Chinese Academy of Agricultural Sciences, Harbin, China

**Keywords:** Cyclooxygenase-2 inhibition, ExPEC, autophagy, cell death, IL-10

## Abstract

Extraintestinal pathogenic *Escherichia coli* (ExPEC) is one of the top pathogens responsible for bloodstream infection and severe, often fatal, sepsis. Although the virulence factors and host immune responses to ExPEC infection have been investigated, the responses to a particular ExPEC strain could be very different. In this study, we investigated the mechanisms of Cyclooxygenase-2 (COX-2) up-regulation in influencing the host defenses against infection of ExPEC XM O2:K1:H7. Our results demonstrated that ExPEC XM O2:K1:H7 infection in mouse and RAW264.7 macrophages leads to COX-2 up-regulation, and COX-2 inhibition significantly enhances ExPEC infection. The up-regulation of COX-2 in macrophages was mediated by Toll-like receptor 4 (TLR4) through the activation of p38 and extracellular signal-regulated kinase/Mitogen-activated protein kinase (ERK/MAPK) pathways. Further studies showed that COX-2 inhibition significantly decreased autophagy in macrophages during ExPEC XM O2:K1:H7 infection. Autophagy inhibition significantly enhanced, while induction reduced ExPEC XM O2:K1:H7 survival in macrophages. In addition, COX-2 inhibition significantly increased macrophage cell death during ExPEC XM O2:K1:H7 infection and increased the expression of anti-inflammatory cytokine interleukin-10 (IL-10). Our results indicate that COX-2 up-regulation benefits host defense against ExPEC XM O2:K1:H7 infection by increasing autophagy in macrophages and by reducing IL-10 expression and macrophage cell death during ExPEC infection.

## Introduction

Extraintestinal pathogenic *E. coli* (ExPEC) are the major gram-negative pathogens responsible for a spectrum of diseases in humans and animals, including septicemia, neonatal meningitis, and urinary tract infections. ExPEC are responsible for 70–95% of urinary tract infections and 20–40% of neonatal meningitis (Russo and Johnson, 2003; George and Manges, 2010). All the diseases caused by ExPEC infection in humans can develop into septicemia, which is the major cause of mortality (Kirkby et al., 2012; Poolman and Wacker, 2016). Conservative estimates show that *E. coli* is responsible for 17% of cases of severe sepsis (Russo and Johnson, 2003). The costs per patient with sepsis are between $27,461 and $32,421 (Arefian et al., 2017) and account for annual costs to the US health system of more than $20 billion (Torio and Andrews, 2006). The emergence of multidrug resistance in ExPEC underscores the urgent need to develop alternative treatments to manage the systemic infection caused by ExPEC (Alhashash et al., 2013).

*E. coli*-related sepsis is a complex systemic disease. Rapid removal of bacteria by the host with appropriate methods is the key to its successful management (Vincent, 2016). Studies on the response of host innate immunity to ExPEC infection may provide new insights into molecular pathogenesis of ExPEC and allow the development of novel therapeutic strategies against *E. coli-*related sepsis. Previous transcriptome analysis of the bladder response to ExPEC identified that IL-10 synthesis benefits the host for defense against *E. coli* (Duell et al., 2012). In addition, the C5aR1 protein, which is involved in complement pathway activation, was shown to contribute to the pathogenesis of ExPEC mediated chronic kidney infection (Choudhry et al., 2016). Cyclooxygenase-1 (COX-1) and COX-2 are the two major prostaglandin H synthases that are responsible for the production of prostaglandins (PGs), involved in regulating immune and inflammatory responses. COX-1 is basally expressed in many cells under normal conditions and is considered to maintain the PG homeostasis, whereas COX-2 is predominantly expressed in cells stimulated with inflammatory mediators (Medeiros et al., 2012; Agard et al., 2013). Despite the fact that COX-2 is well known to be involved in regulating the host immune response against bacterial infection, the outcomes of previous studies on COX-2 inhibition during different bacterial infections remain controversial. COX-2 inhibition was demonstrated to enhance the bacterial clearance in *Pseudomonas aeruginosa* and *Burkholderia pseudomallei* infected mice. Inhibition of COX-2 also protected mice against cystitis caused by ExPEC infection (Hannan et al., 2014). However, the mortality of cecal ligation and puncture (CLP) treated mice was increased in the absence of COX-2 (Fredenburgh et al., 2011). COX-2 up-regulation in normal human keratinocytes may benefit the clearance of *Staphylococcus aureus* (Bernard and Gallo, 2010). The precise underlying mechanisms vary in different species of bacterial pathogens and require further investigation.

Autophagy is a cellular process that contributes to the degradation of cytoplasmic proteins and organelles. Through this innate immune response system, bacteria are ingested and delivered to the lysosome for degradation (Huang and Brumell, 2014). The defects in autophagy impaired the ability of epithelial cells and macrophages to control adherent invasive *E. coli* replication. However, increasing evidence indicates that bacteria have developed sophisticated mechanisms to overcome the autophagy process (Campoy and Colombo, 2009). Autophagy related gene deficiency was reported to confer protection in ExPEC O18:K1:H7 mediated urinary tract infection *in vivo* (Wang et al., 2012). Recently another study demonstrated that COX-2 contributes to host defense against mycobacterial infection by promoting autophagy formation in *Mycobacterium tuberculosis* infected macrophages. COX-2 mediated PG production was also demonstrated to be involved in inducing autophagy in various physiological processes (Kar et al., 2009; Choi et al., 2014; Pelissier-Rota et al., 2015). However, the function of autophagy in ExPEC infection may vary depending on the strain and pathotype, and the association between ExPEC-induced COX-2 expression and autophagy formation has not yet been elucidated (Xiong et al., 2018).

Our previous results showed that COX-2 was among the top 10 genes that were up-regulated in mouse spleen infected with ExPEC XM O2:K1:H7. In this study, we confirmed the role of COX-2 inhibition in ExPEC XM O2:K1:H7 bloodstream infection and identified the pathways involved in inducing COX-2 up-regulation. Our research results indicate that COX-2 up-regulation may benefit the host for the clearance of ExPEC XM O2:K1:H7 by inhibiting macrophage death, IL-10 synthesis, and promoting autophagy formation.

## Materials and Methods

### Animal Infection Assay

Specific pathogen-free six-week-old male and female BALB/c mice were purchased from the Comparative Medicine Center of YangZhou University and housed in specific pathogen-free conditions. To rule out the effect of gender, equal numbers of male and female mice were used in this study. For the infection challenge, mice were inoculated by intraperitoneal injection with 1 × 10^6^ CFU of bacteria resuspended in 100 μL sterile phosphate buffered saline (PBS). NS398 (Selleck, 15 mg/kg) inhibitor or DMSO vehicle treatment were administered at 4 h prior to and 4 and 8 h after inoculation. Mice received 100 μL NS398 dissolved in DMSO or DMSO vehicle by intraperitoneal injection. In the survival experiment, mice were observed for 72 h for signs of morbidity and mortality. Mice were euthanized the nearing endpoint. Euthanasia endpoints used in this study included loss of 20% body weight, hunched posture, and decreased movement, or response to stimuli, or paralysis. In bacterial load assessment assay, the blood, liver, lung, spleen, brain, and kidney of mice were collected at 24 h post infection, and bacterial numbers were evaluated by plating 10-fold serial dilutions of blood, liver, lung, spleen, brain, and kidney homogenates on MacConkey Agar.

### Bacterial Strain, Culture Conditions, and Cell Culture

The ExPEC XM (O2:K1:H7), belonging to the phylogenetic *E.coli* reference (ECOR) group B2, has zoonotic potential and causes septicemia in both mammalian and avian models (Ma et al., 2018; Zhang et al., 2019). The strain was cultured in Luria Bertani (LB) medium at 37°C. The mouse RAW264.7 macrophage (ATCC TIB-71) cell line was purchased from ATCC. The cells were cultured in Dulbecco’s modified Eagle’s medium (DMEM, Hyclone^TM^, GE) supplemented with 10% fetal bovine serum (Gibco, United States).

### Bone Marrow-Derived Macrophages (BMDMs) Isolation Assay

Mouse BMDMs were collected and differentiated *in vitro* from bone marrow cells isolated from femurs and tibias of equal numbers of male and female BALB/c mice (Zhang et al., 2008). During the process, erythrocyte contamination were removed by red blood cell lysis buffer (Solarbio, Beijing, China). Bone marrow cells were pelleted by centrifugation at 500 × *g*, 8 min, 4°C. Cell pellets were resuspended in DMEM containing 10% fetal bovine serum (FBS), 1% penicillin-streptomycin solution, and 20 ng/mL M-CSF (R&D Systems, United States) and cultured in sterile plastic petri dishes. On day 3, another 5 mL of medium was added to each dish. At day 5, cells were washed three times with PBS and harvested by using trypsin.

### Macrophages Infection Assay

Monolayers of both RAW264.7 and BMDM cells were infected with ExPEC XM O2:K1:H7 at indicated multiplicity of infection (MOI) for 1 h. Immediately after the addition of bacteria, the plates were centrifuged for 5 min at 400 × *g* at 25°C to facilitate infection. After incubation at 37°C in 5% CO_2_ for 1 h, extracellular bacteria were killed with 200 μg/mL gentamicin for 1 h. Cells were further incubated with medium containing 25 μg/mL gentamicin to the exclusion of extracellular bacteria. The time of the second addition of gentamicin was defined as time 0. At indicated times, cells were collected to assess the mRNA and protein level of COX-2 by qRT-PCR and western-blot.

Intracellular bacterial killing assays were conducted according to the previous report with slight modification. RAW264.7 cells were treated with rapamycin (MCE, 10 μM) or 3-methyladenine (MCE, 10 μM) for 2 h, and then infected with ExPEC XM O2:K1:H7 at MOI 10 for 1 h. After incubation at 37°C in 5% CO_2_ for 1 h, extracellular bacteria were killed with 200 μg/mL gentamicin for 1 h. Cells were then further incubated with a medium containing 25 μg/mL gentamicin to the exclusion of extracellular bacteria. The time of the second addition of gentamicin was defined as time 0. At 0 h, 3 h, and 6 h post infection, cells were washed with PBS three times and lysed with sterile 0.1% Triton-100 for 15 min. The number of internalized bacteria was determined by plate counting. These experiments were performed in triplicate with three biological repetitions.

### Inhibition Assay

COX-2 inhibitor NS398 (50 μM), p38 MAPK inhibitor SB202190 (FHPI) (10 μM), and ERK1/2 MAPK inhibitor SCH772984 (10 μM), was purchased from Selleck Chemicals (Houston, United States). TLR4 inhibitor TAK-242 (10 μM) was purchased from APEXBIO Technology (Houston, United States). Cells were separately pretreated with these drugs at the indicated concentrations for 1 h at 37°C. At the time of the second addition of gentamicin, the inhibitors were also added, and the point was defined as time 0. Cells treated with TAK-242 were collected at 1 h after infection to detect the protein level of phosphorylated and unphosphorylated ERK1/2 MAPK and p38 MAPK, and at 6 h after infection to determine the COX-2 expression level. ERK1/2 inhibitor SCH772984 and p38 MAPK inhibitor SB202190 (FHPI) treated cells were collected at 6 h after infection to detect COX-2 expression level. NS398 treated cells were collected at 6 h and 9 h after infection to detect reactive oxygen species (ROS), cell death, and cytokine expression levels. NS398 treated cells were collected at 6 h after infection to investigate the LC3 expression level. These experiments were performed in triplicate with three biological repetitions.

### ROS Detection Assay

The level of intracellular ROS was detected by the Reactive Oxygen Species Assay Kit (Beyotime Biotechnology, China, S0033) according to the manufacturer’s instruction. Cells were incubated with DCFH-DA for 20 min in the dark. The level of fluorescent DCF, which reflects the level of ROS, was detected by a fluorometric microplate reader at an excitation wavelength of 488 nm and emission wavelength of 525 nm. These experiments were performed in triplicate with three biological repetitions.

### Western Blot

Cells were washed once with PBS, and were then lysed with Cell Lysis Buffer supplemented with PMSF. Cell Lysate mixed with loading buffer was subjected to SDS-PAGE. Proteins were transferred to a PVDF membrane (Millipore, United States). The membrane was blocked with 5% Non-fat Dry Milk at 37°C for 2 h and further incubated with specific antibodies, Cox2 (D5H5) XP^®^ Rabbit mAb (CST), GAPDH (D16H11) XP <^®^ Rabbit mAb (CST), phospho-p44/42 MAPK (Erk1/2) (Thr202/Tyr204) (D13.14.4E) XP <^®^ Rabbit mAb (CST), p44/42 MAPK (Erk1/2) Antibody (CST), phospho-p38 MAPK (Thr180/Tyr182) (D3F9) XP <^®^ Rabbit mAb (CST), p38 MAPK (D13E1) XP <^®^ Rabbit mAb (CST), and Anti-LC3B antibody produced in rabbit (Sigma) overnight at 4°C. After washing three times with TBST, the membrane was incubated with HRP-linked anti-rabbit IgG antibody (1:2000, CST) for 1 h at 37°C. After washing with TBST, the membrane was incubated with Chemiluminescence Luminol Reagent and analyzed with the Azure c300 Gel Imaging System.

### qRT-PCR

The quantitative reverse transcription PCR (qRT-PCR) was used to verify the differentially expressed genes selected with gene-specific primer (Supplementary Table 1). The total RNA isolated above was reverse transcribed into cDNA using PrimeScript^TM^ RT reagent Kit with gDNA Eraser kit (TaKaRa). qPCR was conducted on the Applied Biosystems QuantStudio 3 system using SYBR <^®^ Premix Ex Taq^TM^ II (TaKaRa). The relative expression level of mRNA was normalized to GAPDH in each sample. The relative fold change was calculated by the threshold cycle (2^–ΔΔ*CT*^) method (Livak and Schmittgen, 2001). These experiments were performed in triplicate with three biological repetitions.

## Elisa

The TNF-α, IL-6, and IL-10 protein levels in each supernatant were determined by the ELISA kits (Multisciences Biotech, Hangzhou, China) according to the manufacturer’s instructions. These experiments were performed in triplicate with three biological repetitions.

### Statistical Analyses

All statistical analyses were performed with Graphpad Prism version 7. Bacterial loads of ExPEC XM O2:K1:H7 in BALB/c mice blood, liver, lung, spleen, and brain as well as the survival time of mice treated with DMSO vehicle and NS398 were compared using unpaired *t* tests. The ROS level and cell death levels in macrophages were compared using two-way ANOVA. Cytokine mRNA and protein levels in macrophages were compared using one-way ANOVA. The number of intracellular viable bacteria in RAW264.7 macrophages was compared using unpaired *t* tests, where the ExPEC XM O2:K1:H7 infected group severed as control. A *P*-value <0.05 was defined as statistically significant.

## Results

### Cox-2 Inhibition Enhanced ExPEC XM O2:K1:H7 Infection in Mouse

**FIGURE 1:**
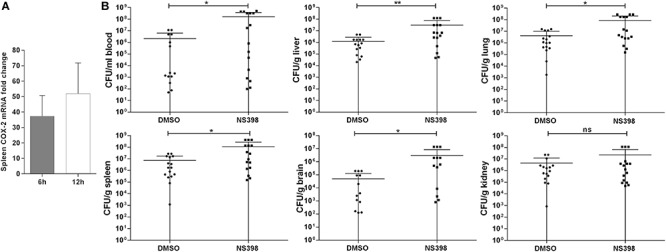
COX-2 is up-regulated in ExPEC XM O2:K1:H7-infected mouse spleen and COX-2 inhibition increases ExPEC XM O2:K1:H7 burden in the blood, liver, lung, spleen, and brain. **(A)** The mRNA expression level of COX-2 in mouse spleen after intraperitoneal injection with 1 × 10^6^ CFU of ExPEC XM O2:K1:H7 was analyzed using quantitative real-time reverse transcription PCR (qRT-PCR). The qRT-PCR results were normalized to the housekeeping gene GAPDH. **(B)** Bacterial burden in mice treated with NS398 or DMSO after intraperitoneal infection with 1 × 10^6^ ExPEC XM O2:K1:H7 was analyzed. Bacteria re-isolated from blood and homogenates of liver, lung, spleen, brain, and kidney at 24 h post infection were assessed by plate counting assay. Statistical significance was determined by using unpaired *t* test (*P < 0.05, **P < 0.01).

Our transcriptome study showed that ExPEC XM O2:K1:H7 infection significantly up-regulated COX-2 expression in mouse spleen. The mRNA level of COX-2 was confirmed to be up-regulated at 6 hpi and 12 hpi in the ExPEC XM O2:K1:H7-infected spleen using quantitative real-time reverse transcription PCR (qRT-PCR) (Figure 1A). To further explore the function of COX-2 in ExPEC XM O2:K1:H7 infection, bacterial loads of ExPEC XM O2:K1:H7 in BALB/c mice treated with NS398, a specific COX-2 inhibitor, or DMSO vehicle were assessed. Compared with the DMSO treated group, significantly more CFU of ExPEC XM O2:K1:H7, were isolated from blood, liver, lung, spleen, and brain in NS398 treated mice, but no significant difference was observed in the kidney (Figure 1B). Further studies were conducted to explore the effects of COX-2 inhibition on survival in mice with an inoculation of 1 × 10^6^ CFU bacteria. No significant difference in survival time existed between mice treated with DMSO vehicle and NS398 (Supplementary Figure 1).

### ExPEC XM O2:K1:H7 Infection Leads to COX-2 Up-Regulation in Macrophages

**FIGURE 2:**
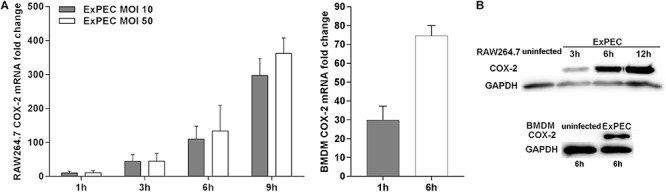
ExPEC XM O2:K1:H7 infection leads to COX-2 up-regulation in macrophages. **(A)** The mRNA expression level of COX-2 in RAW264.7 and BMDM macrophages under ExPEC XM O2:K1:H7 infection were analyzed by qRT-PCR. qRT-PCR results were normalized to the housekeeping gene GAPDH. **(B)** The protein expression level of COX-2 in RAW264.7 and BMDM macrophages after ExPEC XM O2:K1:H7 infection at MOI 10 was analyzed by western-blot. Western-blot results were also analyzed with GAPDH as a loading control. The qRT-PCR represented the mean ± SD of three independent experiments.

We assessed the expression level of COX-2 in ExPEC XM O2:K1:H7-infected macrophages *in vitro*. The bacterial load of ExPEC XM O2:K1:H7 in RAW264.7 and BMDM cells was evaluated (Supplementary Figure 2). The expression level of COX-2 in RAW264.7 and BMDM cells infected with or without ExPEC XM O2:K1:H7 was assessed (Figure 2). Results showed that the mRNA of COX-2 was significantly increased in ExPEC XM O2:K1:H7-infected RAW264.7 and BMDM in a time-dependent manner (Figure 2A). In ExPEC XM O2:K1:H7-infected RAW264.7 macrophages, the mRNA expression level of COX-2 was higher in MOI 50. The protein of COX-2, which could not be detected in uninfected cells, was also significantly increased in ExPEC XM O2:K1:H7-infected RAW264.7 and BMDM cells in a time-dependent manner (Figure 2B).

### ExPEC XM O2:K1:H7 Infection Upregulated COX-2 Expression in Macrophages Through Activating TLR4-Mediated p38 and ERK1/2 MAPK Pathway

**FIGURE 3:**
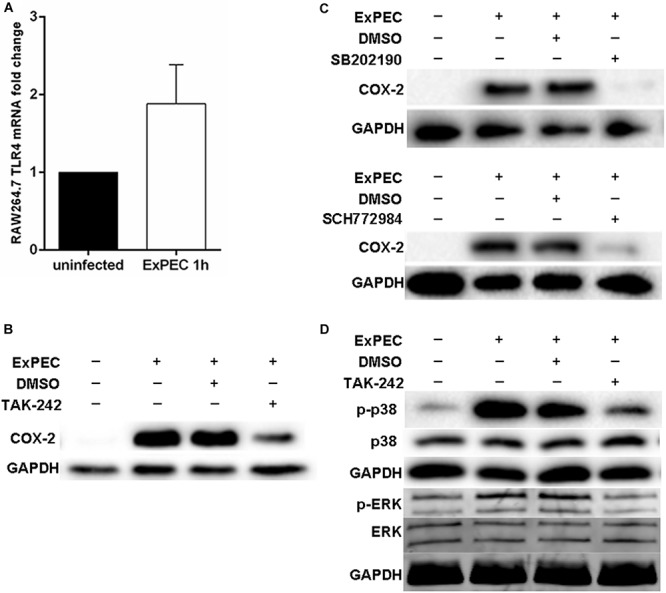
COX-2 up-regulation in ExPEC XM O2:K1:H7-infected macrophages through TLR4 mediated p38 and ERK1/2 MAPK pathway. **(A)** The TLR4 mRNA level of macrophages with or without ExPEC XM O2:K1:H7 treatment at 1 h was assayed by qRT-PCR. **(B)** Western blot analysis of COX-2 expression levels from cell lysates of RAW264.7 macrophages treated with or without TLR4 inhibitor (TAK-242) accompanied by ExPEC XM O2:K1:H7 infection for 6 h. **(C)** Western blot analysis of COX-2 expression levels from cell lysates of RAW264.7 macrophages treated with or without p38 MAPK inhibitor [SB202190 (FHPI)] and ERK1/2 MAPK inhibitor (SCH772984), accompanied by ExPEC XM O2:K1:H7 infection for 6 h. **(D)** Western blot analysis of indicated proteins with specific antibodies from cell lysates of RAW264.7 macrophages treated with or without 10 μM TAK-242, accompanied by ExPEC infection for 1 h.

TLR4 is an important ligand to lipopolysaccharide (LPS) of gram-negative bacteria and plays an important central role in recognizing pathogenic infection (Kawai and Akira, 2009). qRT-PCR results indicated that the mRNA level of TLR4 was significantly up-regulated at 1 h in RAW264.7 macrophages infected with ExPEC XM O2:K1:H7 compared to the control group (Figure 3A). The role of TLR4 in mediating COX-2 up-regulation in ExPEC XM O2:K1:H7-infected macrophages was investigated. Pretreatment with TLR4 inhibitor TAK-242 significantly inhibited COX-2 expression in ExPEC XM O2:K1:H7-infected macrophages RAW264.7, suggesting that TLR4 is involved in COX-2 up-regulation (Figure 3B). TLRs activate p38 and the ERK1/2 MAPK signaling pathway modulating the downstream innate immune response (Kawai and Akira, 2009). Their effects on ExPEC XM O2:K1:H7-induced COX-2 expression was therefore also studied. ExPEC XM O2:K1:H7-infected macrophages RAW264.7 pretreated with p38 inhibitor SB202190 (FHPI) and ERK1/2 inhibitor SCH772984 dramatically decreased the expression of COX-2, and the p38 inhibitor had better inhibitory effect (Figure 3C). To confirm the role of TLR4 in activating p38 and the ERK1/2 MAPK signaling pathway, the phosphorylation levels of p38 and ERK1/2 MAPK were determined by western-blot (Figure 3D). Compared with the uninfected group, RAW264.7 macrophages infected with ExPEC XM O2:K1:H7 showed increased phosphorylation levels. These were suppressed in cells treated with the TLR4 inhibitor.

### COX-2 Inhibition Attenuated Macrophage Autophagy During ExPEC XM O2:K1:H7 Infection and Autophagy Formation Restrained Bacteria Survival in Macrophages

**FIGURE 4:**
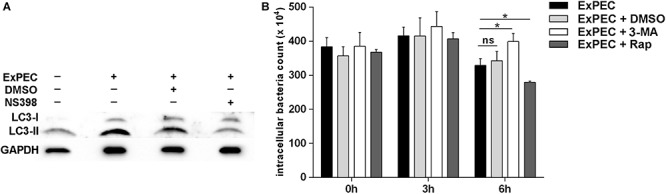
COX-2 inhibition attenuated autophagy in ExPEC XM O2:K1:H7-infected macrophages *in vitro* and autophagy formation contribute ExPEC XM O2:K1:H7 clearance. **(A)** Western-blot analysis of LC3 expression of RAW264.7 macrophages treated with COX-2 inhibitor NS398 or DMSO at 6 h after infection with ExPEC XM O2:K1:H7 at MOI 10. **(B)** RAW264.7 macrophages cells pretreated with autophagy inhibitor 3-methyladenine (3-MA; 10 μM), autophagy inducer rapamycin (rap; 10 μM) and DMSO or PBS vehicle were infected with ExPEC XM O2:K1:H7 at MOI 10, the numbers of intracellular viable bacteria were collected at the indicated times and determined by plate counting assay. Statistical significance was determined by using unpaired *t* test (*P < 0.05).

Autophagy is an efficient cellular process for the host to eliminate pathogens, and COX-2 was reported to be beneficial for the clearance of *M. tuberculosis* by promoting autophagy (Xiong et al., 2018). To explore the effect of COX-2 inhibition on autophagy in ExPEC-infected macrophages, the expression level of the autophagy formation marker LC3, which is converted into LC3-II from LC3-I, was assessed in the study. As shown in Figure 4A, the activated LC3-II was significantly increased in ExPEC XM O2:K1:H7-infected macrophages compared to the uninfected group. The activated LC3-II was significantly decreased in ExPEC XM O2:K1:H7-infected RAW264.7 macrophages that were treated with the COX-2 inhibitor NS398.

To further explore the function of autophagy formation in ExPEC infection, the survival of ExPEC XM O2:K1:H7 in RAW264.7 macrophages treated with autophagy inducer rapamycin (rap) or autophagy inhibitor 3-Methyladenine (3-MA) was monitored. RAW264.7 macrophages treated with autophagy inhibitor 3-MA showed a significantly increased bacterial count at 6 hpi, and conversely, autophagy inducer rap treatment significantly decreased the bacterial count. This indicates that autophagy formation during ExPEC infection reinforces the clearance of bacteria (Figure 4B).

### COX-2 Inhibition Increased Cell Death of Macrophages During ExPEC XM O2:K1:H7 Infection

**FIGURE 5:**
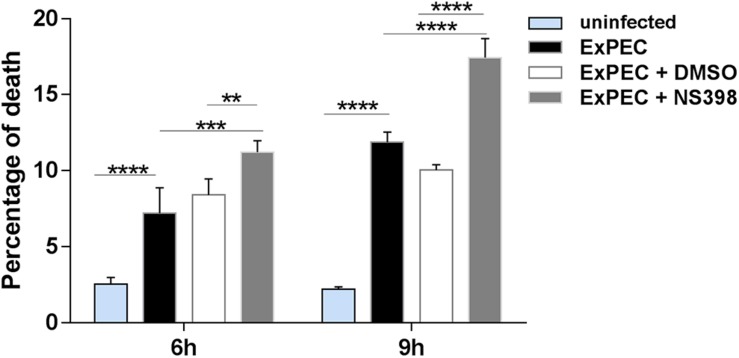
COX-2 inhibition increased cell death of ExPEC XM O2:K1:H7-infected macrophages *in vitro*. RAW264.7 macrophages treated with COX-2 inhibitor NS398 or DMSO vehicle, were infected with ExPEC XM O2:K1:H7 at MOI 10. At 6 h and 9 h after ExPEC XM O2:K1:H7 infection, the cells were stained with 25 μM Propidium Iodide (PI) and cell death level was determined by flow cytometry. Data is shown as the mean ± SEM and represents the results from three independent experiments. Statistical analysis was performed using two-way ANOVA test (**P < 0.01, ***P < 0.001, ****P < 0.0001).

Previous studies have confirmed that COX-2 up-regulation is associated with cell death (Tsujii and DuBois, 1995; Chen et al., 2008). Bacteria were reported to escape from the host by inducing macrophage apoptosis, which benefits its proliferation in the host (Dai et al., 2018). Propidium iodide (PI) staining was conducted to explore the effect of COX-2 inhibition on the cytotoxicity of ExPEC XM O2:K1:H7-infected macrophages. Compared with the uninfected group, cell death levels in the ExPEC XM O2:K1:H7-infected groups were significant higher (Figure 5). Among the ExPEC XM O2:K1:H7-infected groups, cell death rate of RAW264.7 macrophages pretreated with the COX-2 inhibitor NS398 was significantly higher than those pretreated with the DMSO vehicle without COX-2 inhibitor (Figure 5).

### COX-2 Inhibition Did Not Affect Macrophage ROS Production and Inflammatory Cytokines IL-6 and TNF-α Expression Level, but Increased the Expression of Anti-inflammatory Cytokine IL-10

**FIGURE 6:**
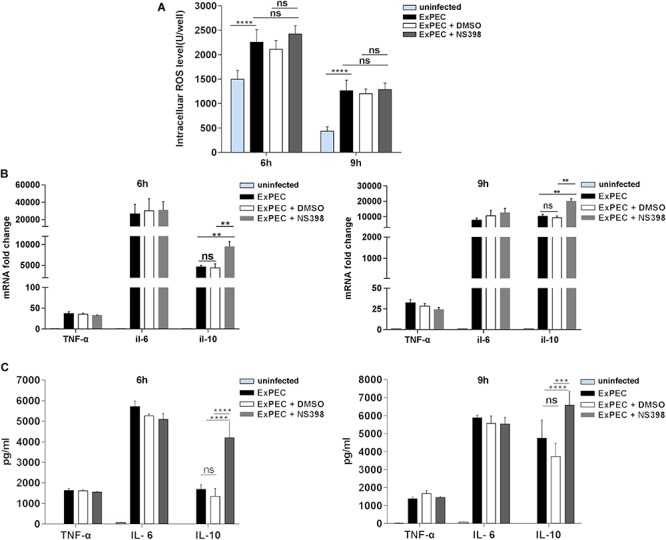
The effect of COX-2 inhibition on the ROS production and inflammatory cytokines expression of macrophages. **(A)** COX-2 inhibition did not affect macrophage ROS production. RAW264.7 macrophages treated with COX-2 inhibitor NS398 or DMSO vehicle, were infected with ExPEC XM O2:K1:H7 at MOI 10. At 6 h and 9 h after ExPEC XM O2:K1:H7 infection, ROS levels of RAW264.7 macrophages were determined with the Reactive Oxygen Species Assay Kit. **(B,C)** COX-2 inhibition did not affect the expression of inflammatory cytokines IL-6 and TNF-α but increased that of anti-inflammatory cytokines IL-10. RAW264.7 macrophages were treated with COX-2 inhibitor NS398 or DMSO vehicle, at 6 h and 9 h after ExPEC XM O2:K1:H7 infection at MOI 10, the cells and supernatants were then harvested for RNA extraction and ELISA assay. The mRNA and protein levels of IL-6, TNF-α, and IL-10 were determined by qRT-PCR and ELISA kits. qRT-PCR results were normalized to the housekeeping gene GAPDH. Data is shown as the mean ± SEM and represents the results from three independent experiments (**P < 0.01, ***P < 0.001, ****P < 0.0001).

ROS is an important component of macrophages involved in bacterial clearance. Based on the fact that COX-2 inhibition promotes ExPEC loads in mice, the level of ROS in ExPEC infected macrophages was studied. The ROS level of ExPEC XM O2:K1:H7-infected RAW264.7 macrophages was significantly higher compared to uninfected macrophages. When treated with COX-2 inhibitor NS398, ROS levels were not changed in the ExPEC XM O2:K1:H7-infected RAW264.7 macrophages (Figure 6A).

Excessive inflammation is an important factor influencing the outcome of sepsis. In this study, we evaluated the effect of COX-2 inhibition on the expression of cytokines. As shown in Figure 6B, in ExPEC XM O2:K1:H7-infected macrophages, those treated with NS398 did not have obvious changes inflammatory cytokines IL-6 and TNF-α expression but had significantly up-regulated anti-inflammatory factor IL-10. Further, ELISA assay also showed that the protein level of IL-10 in ExPEC XM O2:K1:H7-infected macrophages was significantly increased in NS398 treated cells, whereas the protein levels of IL-6 and TNF-α did not change significantly (Figure 6C).

## Discussion

As an important component of the innate immune system, COX-2 is regarded as an enzyme that is absent in normal cells and tissues but is expressed in cells and tissues under pathogen stimulation. COX-2 inhibition during bacterial infection can be a double-edged sword: COX-2 inhibition may benefit the host defense by increasing ROS and nitric oxide (NO) production and regulating inflammatory cytokines expression (Serezani et al., 2007; Goldmann et al., 2010; Agard et al., 2013), while on the other hand, COX-2 inhibition may suppress bacterial clearance through inhibiting autophagy (Xiong et al., 2018). ExPEC mediated chronic and recurrent cystitis, which can develop into sepsis, may also be prevented by inhibiting COX-2 function (Hannan et al., 2014). In this study, we confirmed that ExPEC XM O2:K1:H7 infection significantly increased COX-2 expression in the spleen of infected mice; however, COX-2 inhibition did not reduce but increased bacterial load in the bloodstream. We further confirmed that ExPEC XM O2:K1:H7 infection significantly increased COX-2 expression in macrophages through TLR4 receptor activation in the p38 and the ERK1/2 MAPK pathway. COX-2 inhibition increased macrophage death, IL-10 expression, and decreased autophagy of macrophages, which may be responsible for the failure of COX-2 inhibitor treatment in removing the invading ExPEC *in vivo*. Therefore, the up-regulation of COX-2 expression during ExPEC infection benefits the clearance of the ExPEC in the host bloodstream. This is also observed in cases of CLP treatment and *Staphylococcus aureus* infection, but not *P. aeruginosa* and *B. pseudomallei* infection (Sadikot et al., 2007; Bernard and Gallo, 2010; Fredenburgh et al., 2011; Asakrah et al., 2013).

Autophagy is a well-conserved cellular pathway that is triggered in response to diverse stress conditions. It is required to maintain cellular homeostasis and recycle cellular components (Chun and Kim, 2018). Accumulated evidence has shown that autophagy contributes to innate and adaptive immune pathways and is vital in the host defense against infection by various bacterial pathogens such as *Mycobacterium tuberculosis*, *Salmonella enterica serovar* Typhimurium, and Group A *Streptococcus* (Huang and Brumell, 2014; Xiong et al., 2018). However, autophagy may play an opposite role in the host defense against other bacterial pathogens. A previous study reported that the deficiency of key protein involved in autophagosome maturation, Atb16L1, increased uropathogenic *E. coli* infection *in vivo* (Wang et al., 2012). Therefore, whether autophagy benefits the host or bacterial pathogens varies with particular bacterial species and/or distinct diseases. Our results showed that COX-2 inhibition decreased macrophage autophagy during ExPEC XM O2:K1:H7 infection and autophagy inhibition significantly increased bacterial survival, while autophagy induction significantly decreased bacterial survival in macrophages. These results suggest that COX-2 up-regulation leads to the activation of autophagy in macrophages and autophagy activation facilitates ExPEC XM O2:K1:H7 strain clearance.

NO is an important component involved in bacterial clearance that benefits the host (Flannagan et al., 2015). Our results also indicate that COX-2 inhibition increases the NO level in ExPEC XM O2:K1:H7-infected macrophages. The mRNA expression level of iNOS, which is a major enzyme involved in NO production, is also increased in NS398 treated macrophages with ExPEC XM O2:K1:H7 infection (Supplementary Figure 3). These results were contradictory to that of our *in vivo* test that showed COX-2 inhibition enhanced ExPEC XM O2:K1:H7 infection in mouse. Therefore, its underlying mechanisms remain unknown and require further study.

TLRs are evolutionarily conserved pattern recognition receptors that detect bacterial pathogen-associated molecular patterns, activate the downstream MAPK pathway, and trigger immune responses (Kawai and Akira, 2009). MAPK pathway activation can manipulate multiple cellular functions, including apoptosis, growth, inflammation, and differentiation. ERK1/2, p38, and JNK/MAPK are the major members of the MAPK family (Hommes et al., 2003). Pathways enriched from our previous study of transcriptome response in mouse spleen to ExPEC XM O2:K1:H7 infection indicated that the TLR signaling pathway was activated during ExPEC XM O2:K1:H7 infection. TLR4 is regarded as an important member of TLRs mainly related to recognizing the LPS ligand from gram-negative bacteria (Kawai and Akira, 2009). It was demonstrated that TLR4 is involved in regulating COX-2 expression in RAW264.7 cells and intestinal epithelial cells (Rhee and Hwang, 2000; Fukata et al., 2006). Our results showed that the TLR4 expression was significantly up-regulated in ExPEC XM O2:K1:H7-infected macrophages, and TLR4 inhibitor TAK-242 significantly suppressed phosphorylation of p38 and ERK1/2 MAPK and decreased COX-2 expression. These results suggested that ExPEC XM O2:K1:H7 infection upregulates TLR4 expression, subsequently leading to the activation (increased phosphorylation level) of p38 and ERK1/2 MAPK, and finally resulting in the up-regulation of COX-2 expression. Indeed, the p38 inhibitor [SB202190 (FHPI)] and ERK1/2 inhibitor (SCH772984) dramatically decreased the expression of COX-2 in ExPEC XM O2:K1:H7-infected macrophages in our study. A previous study showed that both TLR4 and ERK1/2, p38, and JNK MAPK pathways were involved in inducing COX-2 expression in type I fimbriated uropathogenic *E.coli* infection (Chen et al., 2011). Additionally, ERK1/2 and p38 MAPK inhibition decreased COX-2 expression in *Salmonella* infected macrophages (Uchiya and Nikai, 2004). Our study demonstrated that infection of ExPEC XM O2:K1:H7 strain lead to up-regulation of TLR4 and induced COX-2 expression through the activation of p38 and the ERK1/2 MAPK pathway.

In our study, COX-2 inhibition increased cell death of macrophages during ExPEC XM O2:K1:H7 infection. Cell death can be the result of three process: apoptosis, pyroptosis, and necrosis (D’Arcy, 2019). COX-2 up-regulation in cancer cells was associated with inhibiting cancer cell apoptosis (Nzeako et al., 2002; Li et al., 2015; Liu et al., 2015). However, in virulent *M. tuberculosis* infected macrophages, COX-2 mediated PGE_2_ secretion may inhibit the necrosis process of macrophages. Considering that heat-inactivated ExPEC did not induce macrophage apoptosis *in vitro* (Rodrigues et al., 1999), but virulent ExPEC caused severe damage to macrophages (Zhuge et al., 2019), it is very likely that COX-2 up-regulation during ExPEC XM O2:K1:H7 infection may inhibit the apoptosis of macrophages to enhance the human immune system.

In summary, we explored the effect of COX-2 in ExPEC XM O2:K1:H7 infection in a mouse model and mouse macrophage cell model using a COX-2 inhibitor. Our results demonstrated that COX-2 inhibition enhanced ExPEC XM O2:K1:H7 infection in both models, suggesting that COX-2 up-regulation in ExPEC XM O2:K1:H7-infected mice benefits the host and plays a vital role in the host’s innate immunity against ExPEC infections. COX-2 up-regulation may enhance autophagy in macrophages during ExPEC XM O2:K1:H7 infection and autophagy may subsequently decrease bacterial survival in macrophages. This may lead to the clearance of bacteria, thereby benefiting the host defense against ExPEC XM O2:K1:H7 infection. COX-2 up-regulation may also benefit the host defense against ExPEC XM O2:K1:H7 infection through down-regulation of the IL-10 expression, a known anti-inflammatory cytokine (Angus and van der Poll, 2013), and reduction of macrophage cell death during ExPEC XM O2:K1:H7 infection. The underlying mechanisms by which COX-2 up-regulation reduces macrophage cell death during ExPEC XM O2:K1:H7 infection require further study.

## Data Availability Statement

All datasets generated for this study are included in the article/Supplementary Material.

## Ethics Statement

All animal experiments were performed according to the guidelines of the Experimental Animal Management Measures of Jiangsu Province and approved by the Laboratory Animal Monitoring Committee of Jiangsu Province (Nanjing, China).

## Author Contributions

HR, FJ, and GL conceived and designed the experiments. HR and XC performed the experiments. HR, FJ, and GL analyzed the data. HR and GL wrote the manuscript.

## Conflict of Interest

The authors declare that the research was conducted in the absence of any commercial or financial relationships that could be construed as a potential conflict of interest.
