# Supplementary material for: Cyclooxygenase-2 Inhibition Reduces Autophagy of Macrophages Enhancing Extraintestinal Pathogenic Escherichia coli Infection
Source: Front Microbiol. 2020 Apr 17;11:708. doi: 10.3389/fmicb.2020.00708 (PMC7180184; doi:10.3389/fmicb.2020.00708)
Supplement: Supplementary file 1 [file Data_Sheet_1.docx]

Supplementary Figures





**Supplementary Figure 1.** **COX-2 inhibition did not change the survival time of ExPEC infected mice.** Survival curve of mice intraperitoneally infected with 1 × 10^6^ CFU ExPEC XM O2:K1:H7 treated with COX-2 specific inhibitor (NS398) or DMSO (vehicle-treated mice). NS398 and DMSO were intraperitoneally administered at 4hpi pre-infection, 8hpi and 12hpi post-infection. Survival time was monitored for 72 hours.


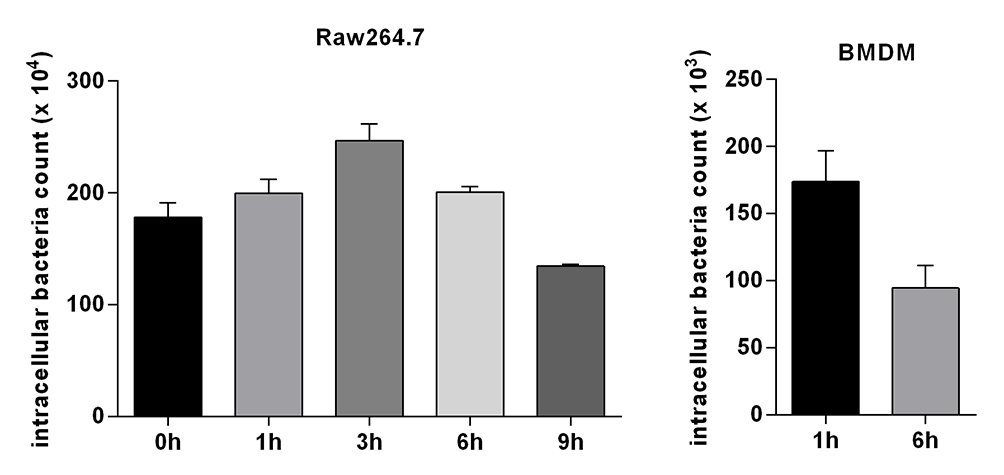


**Supplementary Figure 2.** ***In vitro* bacterial load in assays with RAW264.7 and BMDM macrophages.** RAW264.7 and BMDM macrophages cells were infected with ExPEC XM O2:K1:H7 at MOI 10, the number of intracellular viable bacteria were collected at the indicated times and determined by plate counting assay.


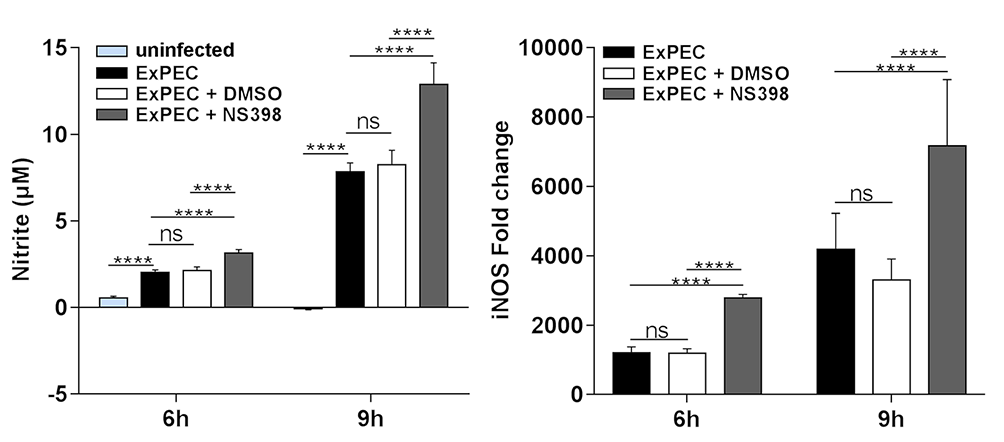


**Supplementary Figure 3.** **COX-2** **inhibition increased the NO level and iNOS expression level of ExPEC XM O2:K1:H7 infected RAW264.7 macrophage.** RAW264.7 macrophages treated with 50μM COX-2 inhibitor NS398 or DMSO vehicle, were infected with ExPEC at MOI 10. At 6h and 9h after ExPEC XM O2:K1:H7 infection, corresponding NO level (as indicated by the nitrite concentration) and iNOS mRNA expression level was measured by using Griess regents and qRT-PCR. Statistical significance was determined by using two – way ANOVA analysis (^****^*P*<0.0001).
