# Supplementary material for: Cyclooxygenase-2 Inhibition Reduces Autophagy of Macrophages Enhancing Extraintestinal Pathogenic Escherichia coli Infection
Source: Front Microbiol. 2020 Apr 17;11:708. doi: 10.3389/fmicb.2020.00708 (PMC7180184; doi:10.3389/fmicb.2020.00708)
Supplement: Supplementary file 2 [file Table_1.docx]

Supplementary Tables

**Supplementary Table 1**

**Table S1 Primers used for qRT-PCR.**

| **Gene symbol** | **Forward Primer Sequence (5’ to 3’)** | **Reverse Primer Sequence (5’ to 3’)** | |
| --- | --- | --- | --- |
| *GAPDH* | AGGTCGGTGTGAACGGATTTG | | TGTAGACCATGTAGTTGAGGTCA |
| *TNF-α* | AAGCCTGTAGCCCACGTCGTA | | GGCACCACTAGTTGGTTGTCTTTG |
| *IL-10* | ACAGCCGGGAAGACAATAAC | | CAGCTGGTCCTTTGTTTGAAAG |
| *IL-6* | CTTCCATCCAGTTGCCTTCT | | CTCCGACTTGTGAAGTGGTATAG |
| *iNOS* | AGGAGGAGAGAGATCCGATTTAG | | TCAGACTTCCCTGTCTCAGTAG |
| *TLR4* | GGGTATTTGACACCCTCCATAG | | CAAGAGTGCTGAGGGAATACAG |
| *COX-2* | GTGCCTGGTCTGATGATGTATG | | TGAGTCTGCTGGTTTGGAATAG |
